# Cultural integration of invasive species

**DOI:** 10.1038/s44185-025-00097-3

**Published:** 2025-06-26

**Authors:** Ivan Jarić, Álvaro Fernández-Llamazares, Zsolt Molnár, Ugo Arbieu, Susan Canavan, Ricardo A. Correia, Franz Essl, Katie L. Kamelamela, Richard J. Ladle, Anne-Claire Maurice, Yves Meinard, Ana Novoa, Martin A. Nuñez, Petr Pyšek, Uri Roll, Valerio Sbragaglia, Ross T. Shackleton, Liron Shani, Kate Sherren, Yael Teff-Seker, Ana Sofia Vaz, Priscilla M. Wehi, Jonathan M. Jeschke

**Affiliations:** 1 Université Paris-Saclay, CNRS, AgroParisTech, Ecologie Société Evolution, Gif-sur-Yvette, France; 2 Biology Centre of the Czech Academy of Sciences, Institute of Hydrobiology, České Budějovice, Czech Republic; 3 Department of Animal Biology, Plant Biology and Ecology (BABVE) and Institute of Environmental Science and Technology (ICTA-UAB), Universitat Autònoma de Barcelona, Cerdanyola del Vallès, Barcelona, Spain; 4 HUN-REN Centre for Ecological Research, Institute of Ecology and Botany, Traditional Ecological Knowledge Research Group, Vácrátót, Hungary; 5 School of Natural Sciences, Ollscoil na Gaillimhe—University of Galway, Galway, Ireland; 6 Biodiversity Unit, University of Turku, Turku, Finland; 7 Helsinki Institute of Sustainability Science (HELSUS), University of Helsinki, Helsinki, Finland; 8 Helsinki Lab of Interdisciplinary Conservation Science (HELICS), Department of Geosciences and Geography, University of Helsinki, Helsinki, Finland; 9 Division of BioInvasions, Global Change & Macroecology, Department of Botany and Biodiversity Research, University of Vienna, Vienna, Austria; 10 School of Ocean Futures, Center for Global Discovery and Conservation Science, Arizona State University, Hilo, HI USA; 11 Institute of Biological and Health Sciences, Federal University of Alagoas, Maceió, AL Brazil; 12 Aix-Marseilles-Université, CNRS, Centre Gilles Gaston Granger (UMR 7304), Aix-en-Provence, France; 13 Estación Experimental de Zonas Áridas, Consejo Superior de Investigaciones Científicas (EEZA-CSIC), Almería, Spain; 14 Czech Academy of Sciences, Institute of Botany, Department of Invasion Ecology, Průhonice, Czech Republic; 15 Department of Biology and Biochemistry, University of Houston, Houston, TX USA; 16 Grupo de Ecología de Invasiones, INIBIOMA, CONICET-Universidad Nacional del Comahue, Bariloche, Argentina; 17 Department of Ecology, Faculty of Science, Charles University, Prague, Czech Republic; 18 Mitrani Department of Desert Ecology, The Jacob Blaustein Institutes for Desert Research, Ben-Gurion University of the Negev, Midreshet Ben-Gurion, Israel; 19 Department of Marine Renewable Resources, Institute of Marine Sciences (ICM-CSIC), Barcelona, Spain; 20 Swiss Federal Institute for Forest, Snow and Landscape Research, Birmensdorf, Switzerland; 21 Centre for Invasion Biology, Department of Botany & Zoology, Stellenbosch University, Stellenbosch, South Africa; 22 Department of Sociology and Anthropology, The Hebrew University, Jerusalem, Israel; 23 School for Resource and Environmental Studies, Dalhousie University, Halifax, Canada; 24 Department of Sociology, University of California, Davis, CA USA; 25 NBI, Natural Business Intelligence, Régia Douro Park, Andrães, Vila Real, Portugal; 26 Centre for Sustainability, University of Otago, Dunedin, New Zealand; 27 Leibniz Institute of Freshwater Ecology and Inland Fisheries (IGB), Berlin, Germany; 28 Institute of Biology, Freie Universität Berlin, Berlin, Germany; 29 Berlin-Brandenburg Institute of Advanced Biodiversity Research (BBIB), Berlin, Germany

**Keywords:** Conservation biology, Invasive species

## Abstract

Many invasive non-native species gradually become embedded within local cultures. Such species can increasingly be perceived by society as familiar or even native elements of the social-ecological system and become an integral part of local cultures. Here, we explore this phenomenon and refer to it as the *cultural integration* of invasive species. Although culturally integrated species can positively contribute to people’s lives and well-being, and provide new or lost ecosystem services, their acceptance can also hinder the ability of conservation managers to successfully manage invasive species by reducing public support for their management. Cultural integration can infringe upon social values and cultural identities, and contribute to the erosion and homogenization of biocultural diversity. It can also modify or displace the cultural uses and values of native species, and may disrupt social-ecological legacies and dynamics. We present the main mechanisms of cultural integration, its drivers and major implications, and provide key recommendations for the management and conservation of biological and cultural diversity.

## Introduction

Biological invasions are a major threat to global biodiversity and lead to profound ecological and socioeconomic impacts^1–3^. Invasive non-native species (henceforth “invasive species”) represent organisms known to have established and spread with negative impacts on biodiversity, local ecosystems and species^3^. Many invasive species also affect nature’s contributions to people (embodying different concepts such as ecosystem goods and services, and nature’s gifts) and good quality of life^3^. After their introduction and establishment, invasive species may become embedded within local cultures through a range of relationships and interactions with people^4,5^, and be increasingly perceived by communities as beneficial components of nature, as familiar species, or even as native elements of the social-ecological system, in a process we refer to as *cultural integration*^6,7^. Cultural integration may be driven or accelerated by processes such as decline or extinction of native species, and human colonization. Over time, invasive species may become increasingly familiar to people and ultimately be perceived as native and/or an integral part of local culture^8–10^.

Perceptions of invasive species are driven by social and ecological processes. These trends can strongly affect societal support for invasive species management^6,7^. Here, we examine the process by which some invasive species become embedded in cultures and societies through collective memory, attention, knowledge, representations, uses, and cultural products. We first screened the literature, which revealed a plethora of different terms related to cultural integration, such as assimilation, incorporation, adoption, naturalization, and percolation of invasive species (Table 1). However, these terms were in most cases left undefined and lacked clarity. Implied meanings of these terms ranged from the use of such species for livelihoods or economy to becoming embedded in culture, customs, rituals and traditions. Furthermore, the same authors often used different terms in different papers, and sometimes interchangeably within the same paper.

**Table 1:** Terms used in the literature to refer to the process of cultural integration, and the contexts suggested

Term	Contexts or definitions suggested	References
Societal acceptance	Becoming accepted as a desirable element of local fauna and flora, through the shifting baseline syndrome	^37^
Adoption	Being included in local livelihoods, cultural practices and traditions, in everyday life, as a food and a resource, in traditional pharmacopoeia, becoming culturally or spiritually important, the symbol of local identity, and part of the local culture	^6–8,28,43,46,48,49,54,94,113^
Assimilation	Being included in a way of life and culture, in cultural practices, culinary tastes, as sociocultural and legal assimilation, acquiring local names, and not being differentiated from native biodiversity	^43,49,114,115^
Cultural assimilation	Being welcomed into a culture, sometimes to the point of becoming a cultural icon	^41^
Naturalization	Being viewed as belonging in a place	^116^
Incorporation	Being included in local systems, cultures, economy, livelihoods, cuisines, pharmacopoeias, rituals, recreational activities, biocultures, and social memory, becoming perceived as an intrinsic part of local ecosystems	^4,7,8,41,64,78,79,117^
Integration	Being included in broader cultural awareness, in local cultures, lifestyles, livelihoods, mythologies and spirituality, becoming cultural icons, important elements of social and spiritual status, of personal, community and sociocultural identities	^6,7,33,41,46,49,53,79,110,115^
Identity integration	Coming to symbolize or encapsulate existing ideas about the defining characteristics of places and people	^53^
Percolation	Being included in the social perception of nativeness	^58^

We propose cultural integration^6,7^ as an umbrella term designed to include and embrace previously used notions, yet we acknowledge that the previously used terms are distinct, and that each of them has been used in a particular context. We understand cultural integration as the meeting point where all these different notions convene and connect to each other. Further, we follow UNESCO’s^11^ definition of culture, as a *“set of distinctive spiritual, material, intellectual and emotional features of society or a social group, that encompasses, in addition to art and literature, lifestyles, ways of living together, value systems, traditions and beliefs*”, which should extend to interactions of members of a society not only with each other but also with their environment, including non-native species. Cultural integration of non-native species in their new locations can be highly beneficial and important for local cultures^12^, but a subset of these introduced species, especially those that become invasive, can also greatly harm local environments, people, and sustainable management. Cultural integration may lead to conflicts between groups of people, impact management implementation^13^, modify or displace the cultural presence and identity of vulnerable native species^14^, and relationships of local communities with other species. Consequently, awareness of cultural integration is important to guide management, in particular for invasion ecologists and practitioners who are not often well informed on social dimensions and processes that can greatly affect control programs.

Here, we aim to synthesize the concept of cultural integration of invasive species, its main mechanisms and drivers, discuss the importance of understanding and tracking cultural integration, and illustrate it with examples of invasive species that are already embedded in local cultures or in the process of becoming so. It is important to note that cultural integration may occur with any non-native species, and not exclusively with those that are invasive. Nevertheless, we focus here on invasive species because the consequences of their cultural integration are particularly relevant for environmental policy and management planning.

## Characteristics and mechanisms of cultural integration

**Fig. 1:**
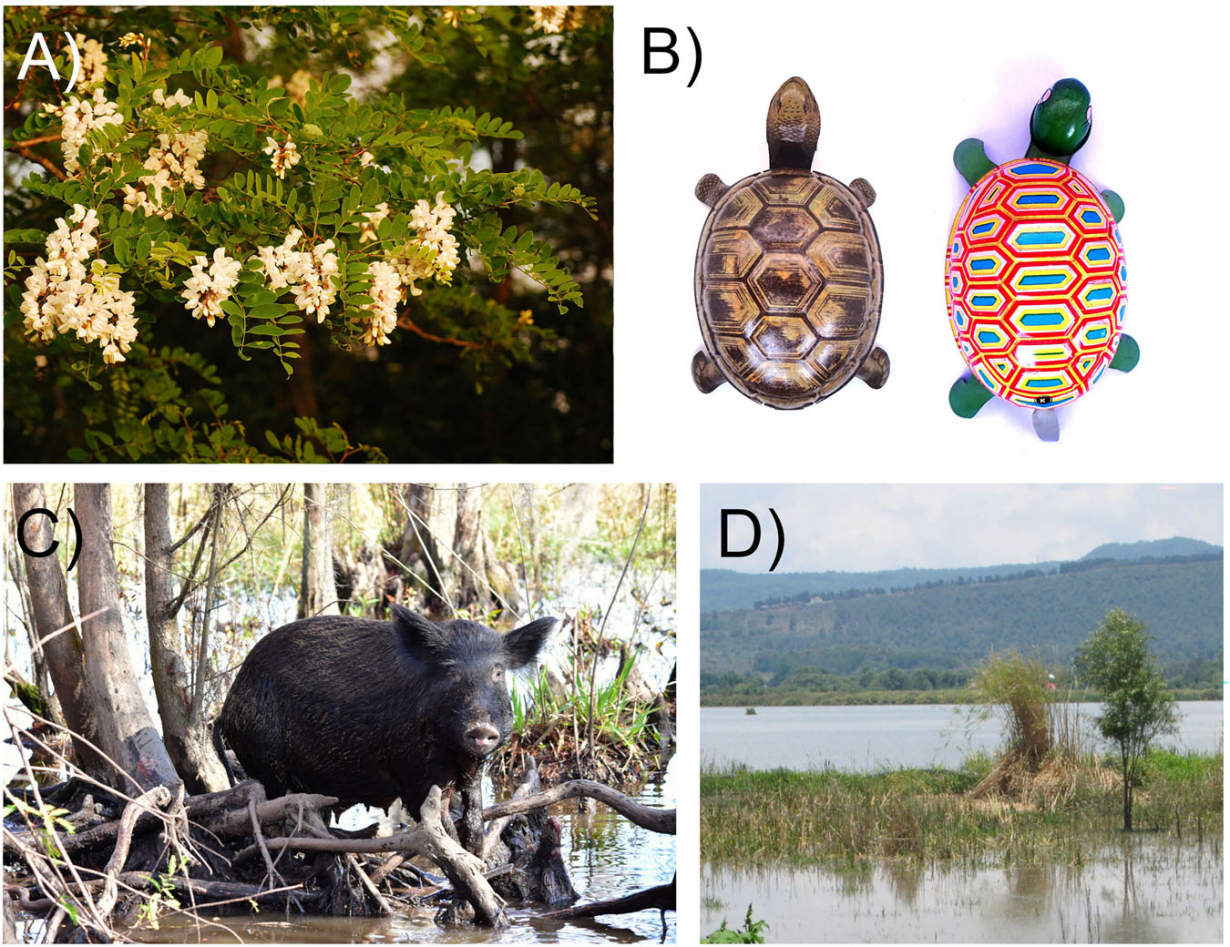
Case examples of cultural integration of invasive non-native species. **A** black locust (*Robinia pseudoacacia*), an invasive non-native species in Hungary that causes conservation problems and is managed in protected areas, is widely perceived as one of the most traditional and useful Hungarian trees (Box 1; Photo: Zsolt Molnár). **B** Japanese tin toy turtles have experienced a notable shift over time from colors dominated by brown and black (left) to those dominated by red, yellow and green (right), which was potentially driven by the dominance of the invasive red-eared slider (*Trachemys scripta elegans*) over native turtle species, and their respective coloration^24^; **C** many feral animals, such as feral pigs (*Sus scrofa*) in the USA^64^, have been incorporated in local culture and economy (Photo: Pedrik); **D** once cattail (*Typha domingensis*) became commodified as a popular resource for handicrafts, local communities in Mexico started to intentionally facilitate its invasion, which is negatively affecting the native California bulrush (*Schoenoplectus californicus*), another culturally valuable wetland plant (Photo: Steven J. Hall)^76^.

The process of cultural integration is an outcome of individual and community exposure and interactions with invasive species^15^^,16,17^, which leads to the incorporation of the species into local cultural identities, practices and norms, and a loss of collective memory about its origin and status^18^. This includes invasive species becoming a source of local identity and pride (e.g., included in place or street names, or local logos)^19,20^, incorporated into cultural practices (e.g., medicinal products and cultural ceremonies)^21^, traditional products and crafts^22,23^, embedded into local folklore, stories, song, and art^6,24^, or into local cuisine^25^ (further examples in Fig. 1, Boxes 1 and 2).

**Fig. 2:**
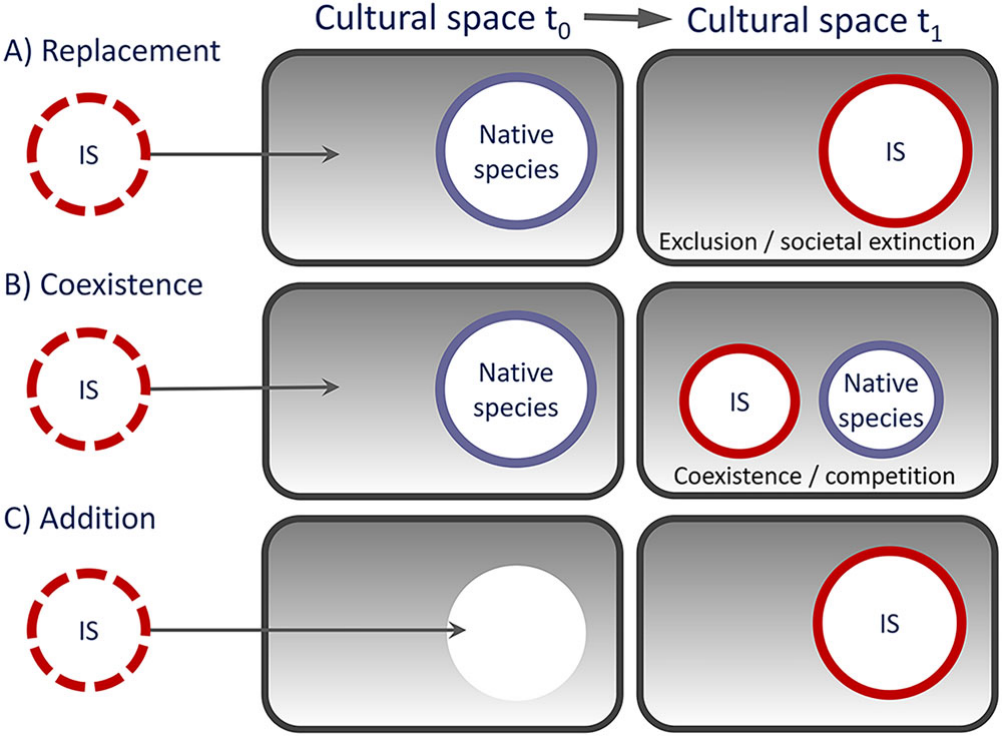
Potential scenarios for interactions of a culturally integrated invasive non-native species (IS) with native species in the cultural space. **A** Replacement of native species by an invasive non-native species within the cultural space, with the exclusion and/or societal extinction^34^ of the native species, and invasive non-native species taking over its roles in livelihoods, customs, and sense of place and identity; **B** coexistence of invasive non-native species and native species in the cultural space, leading to a reduction of the occupied cultural space by the native species due to competition; **C** addition of invasive non-native species in the cultural space that was previously vacant, either through establishment of novel human–nature interactions and cultural practices, or by occupying space of a previously extirpated species. The three archetypes represent only the main types of outcomes, and there are many other possible alternative scenarios and their combinations.

The integration of a non-native species allows it to inhabit an existing or a novel “cultural niche” (see Glossary in Supplementary Table 1) within “cultural space”^26^ (Fig. 2). For some people, human–nature relationships and commitments extend to species within their territories regardless of their native or non-native status^27,28^. While scientific evidence is used to determine species’ native vs. non-native identity^29^, public perceptions of what is native or non-native are largely a result of fluid and highly dynamic social constructions, which arise from species’ ecology, mental representations, and socioeconomic contexts^30–32^. Furthermore, species that gradually acquire a “culturally native” status are often disjoined from their biogeographic status^33^.

Exposure to invasive species can be direct, through people’s daily lives, and activities such as harvesting. However, exposure is often indirect through vicarious experiences based on virtual exposure to various physical or digital records from the literature, arts, and especially through the media, including social media^34^ and parasocial relationships with social media influencers^35^. Vicarious experiences based on virtual exposure, without direct sensory contact with the species^34^, are often based on highly stylized or homogenized species representations^36^, which can be especially effective in influencing people’s attitudes towards invasive species, for example by presenting species as endearing or charismatic^37^. In addition, such mediated experiences can occur regardless of a species’ local presence and status. Mediated experiences can initiate cultural integration before the species is even introduced, often before any direct interactions with the species. The process of cultural integration can also be actively initiated or facilitated, for example, by promoting the social or economic value ascribed to invasive species (e.g., the campaign by the Illinois Department of Natural Resources to rebrand the invasive Asian carp species as “Copi”, to make them more appealing and marketable as food)^38,39^.

The process of cultural integration can be facilitated and intensified by, but also contribute to, the shifting baseline syndrome, i.e., the gradual change in expectations of what people consider to be a “normal” or desirable state of the environment. This can happen due to a lack of experience, memory, or knowledge about past conditions^40^. Consequently, invasive species that are present over a long time period may no longer be perceived by people as invasive or non-native, but as an original and even desirable part of local fauna, flora, and culture^24,37,41,42^. Considering the prevalence of long-established invasive species in many places, it is likely that this process has over time substantially altered perceptions of nature and historical memory^10,43^. For example, many North American Indigenous communities have strong traditions associated with horses (*Equus ferus*), though scientific records suggest horses were introduced there in the 16th century by European colonizers^44^ after their extinction in the Americas about 10,000 years ago.

Cultural integration can occur within a single human generation^16,24,41^. Once integrated, invasive species can have their cultural status amplified over time, sometimes to the point of becoming iconic and embodying cultural, spiritual, or symbolic values^6,31,43^. Nevertheless, it is important to note that, as with native species, many invasive species never become socially and culturally present. This is typically the case for uncharismatic, small, cryptic, or inaccessible species, such as invertebrates (particularly those living underwater or belowground), fungi, and microorganisms^45^ or those that do not provide tangible or intangible benefits and/or exert high impacts^46^.

Once an invasive species is culturally integrated in its new range, it may either occupy “cultural niches” that were previously vacant or, alternatively, it can coexist or sometimes exclude and replace certain native species from their cultural niche (Fig. 2). Through partial or full cultural replacement of native species, invasive species can become “cultural substitutes” for native species in the role the latter play in people’s livelihoods, customs, and sense of place and identity^8^.

Box 1 The cultural integration of black locust in Hungary: a highly controversial, culturally nativized and beloved, invasive non-native tree species “*Why don’t you like this traditional akác?*”—an old woman asked a local conservationist.

Black locust (*Robinia pseudoacacia*), native to North America, was introduced to Hungary in 1710, and became widespread by 1895 after large-scale promotion of the species. Black locust filled an almost empty niche in the by then almost treeless lowlands of Hungary. It became an important source of the economy, with half of the EU plantations located in the country. The species was declared as harmful to biodiversity and invasive in 2009. Pushed by foresters and beekeepers, and widely supported by the public, the tree and its honey attained the status of ‘Hungarikum’, as an element of unique value for the country, and thus entered the political arena and public discourse in 2014. A “*Robinia* Coalition” was founded to lobby for black locust in Hungary and in the EU, to prevent its inclusion in the EU invasive species list. A representative survey in the 1990s showed that it is widely considered “*the most Hungarian tree species*”^113,118^.

Black locust is useful for many people, with 12 identified services and three disservices associated with the species. A fifth of the Hungarian forests consists of black locust, but it is also common among arable fields, along roads, and in small woods around farms. This quickly growing hardwood tree provides high-quality timber, honey (provides half of all honey produced in Hungary), excellent firewood, improves soil, prevents erosion, fixes sand dunes, and is also used for medicinal purposes and as a fodder. Black locust is considered “environmentally friendly”, because no chemical treatment is needed for its outdoor use (e.g., as street furniture) due to its resistance to insects and fungi^113,118^.

There is a high level of awareness and knowledge of black locust in Hungary, with widespread personal relationships and positive attitudes. It has become a cultural keystone species and attained local symbolic value, and is often mentioned in poems and songs. Black locust is regarded as native by most local villagers, even by the traditional knowledge holders, because “*it was already widespread in my childhood*”. Even those people who know that it is non-native regard it as an intrinsic and desirable part of local landscapes. Black locust is generally regarded as “nativized”, especially by foresters who even feel a responsibility towards it and cultivate it, with many cultivars selected. Some forest types, marginal arable lands and abandoned pastures were often reforested with black locust in hilly areas and lowlands, which is a missed opportunity for increasing forest cover with native trees. On the flip side, black locust is regarded as a harmful invasive species by ecologists and conservationists. Black locust can survive in naturally non-forested habitats and replace native vegetation and associated biodiversity. As a result of the benefits and despite the impacts of the tree, there is a strong public opposition to invasive control, particularly as local people have limited understanding about the harms black locust causes to native biodiversity^113,118^.

Many people argue that without black locust, the Great Hungarian plain would be “characterless”^113,118^. This view shows that black locust covers the pre-industrial knowledge of the landscape, as a manifestation of a shifting baseline syndrome. Black locust may have contributed to the erosion of local, traditional knowledge, but possibly of only specific tree species and only in some regions where it became the almost mono-dominant wood source, because other species became less needed, and thus less known. There may be intergenerational differences in the perception of black locust, as the understanding of its invasiveness is increasing^113,118^.

**Figure:**
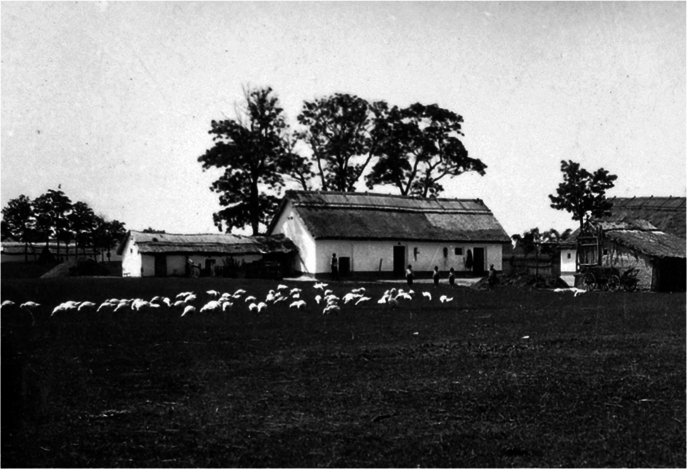
Traditional farm in the Hungarian Great Plain in 1930s, with a stand of black locust (*Robinia pseudoacacia*) visible in the background.

Box 2 Cultural integration of the prickly pear cactus in different social-ecological settings Prickly pear cactus (*Opuntia ficus-indica*) has been introduced into numerous arid and semi-arid areas globally as an ornamental plant, as well as for food and fodder. It has since been adopted into many cultures in Africa, Asia and Europe, particularly after densities and spread were stabilized with effective biological control. For example, in the Eastern Cape of South Africa, collecting and selling fruits from wild prickly pear cactus (commonly named *Itolofiya* in isiXhosa) populations represents an important local livelihood strategy for rural poor communities in the area. Products made from the fruit can be found in many local artisanal stores, which are also present in local recipe books (for jams, drinks and desserts)^119,120^. In arid communities in the north of South Africa (Kalahari region), prickly pear cactus has an important role as a hedge plant, and as a fodder plant used during dry months^121^. In some cases, the social memory of its origins has been forgotten, and it is even depicted in tapestries of local San oral histories and customary law (https://bushmanheritagemuseum.org/the-xam-bushmen/).

In Europe, prickly pear cactus has gone through cultural integration as well. For example, in Sicily (Italy), prickly pear cactus (commonly named *fico d'india* with various spellings) can be found in most gardens and is an important food and economic resource. Local traditions have even developed over time: *“According to tradition, especially that of eastern Sicily, the August harvested fruits are sun-dried and consumed during winter”*, and contracted prickly pear juice “*may be mixed with wheat flour and chopped almonds to prepare a kind of traditional cake called “mustazzol*”^122^. It has become a local symbol of the island and subsequently been incorporated into traditional arts and crafts and is also found in numerous local hotel, restaurant and shop names on the island. In many countries such as Algeria, Cyprus, Egypt, India, Portugal and Romania, prickly pear cactus is depicted on national stamps, showing cultural integration in many regions globally.

**Figure:**
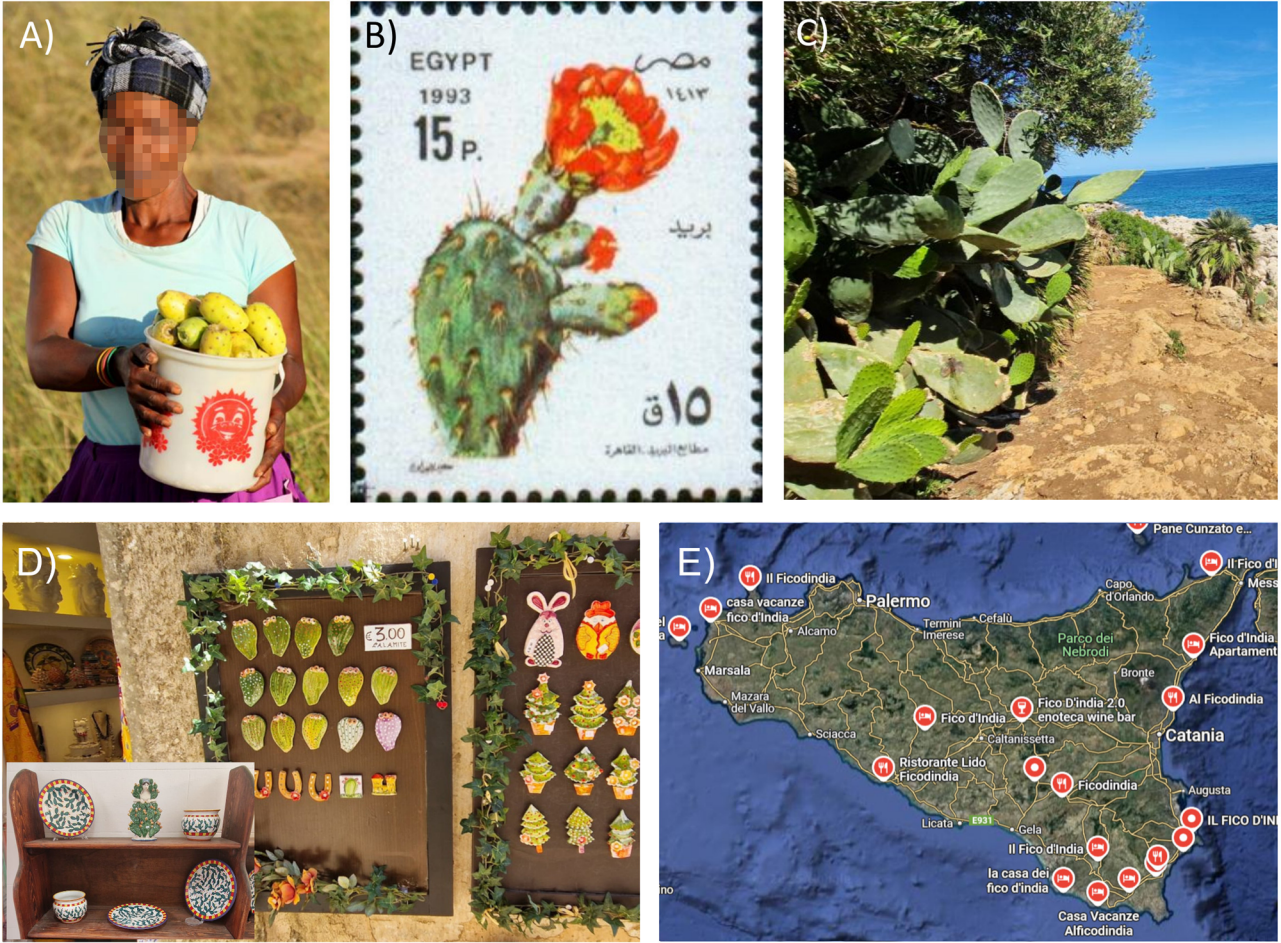
Images depicting the cultural integration of prickly pear cactus (*Opuntia ficus-indica*) in various regions globally. **A** An isiXhosa woman collecting prickly pear fruits in South Africa (Photo: Ross Shackleton); **B** a stamp with prickly pear cactus from Egypt; **C** prickly pear cactus on a popular touristic beach in Sicily (Photo: Ross Shackleton); **D** local pottery in Sicily with prickly pear cactus cladodes (Photo: Ross Shackleton); **E** locations of numerous bars, restaurants and hotels named after prickly pear cactus in Sicily (Google Maps).

## Factors driving the process of cultural integration of invasive species

**Fig. 3:**
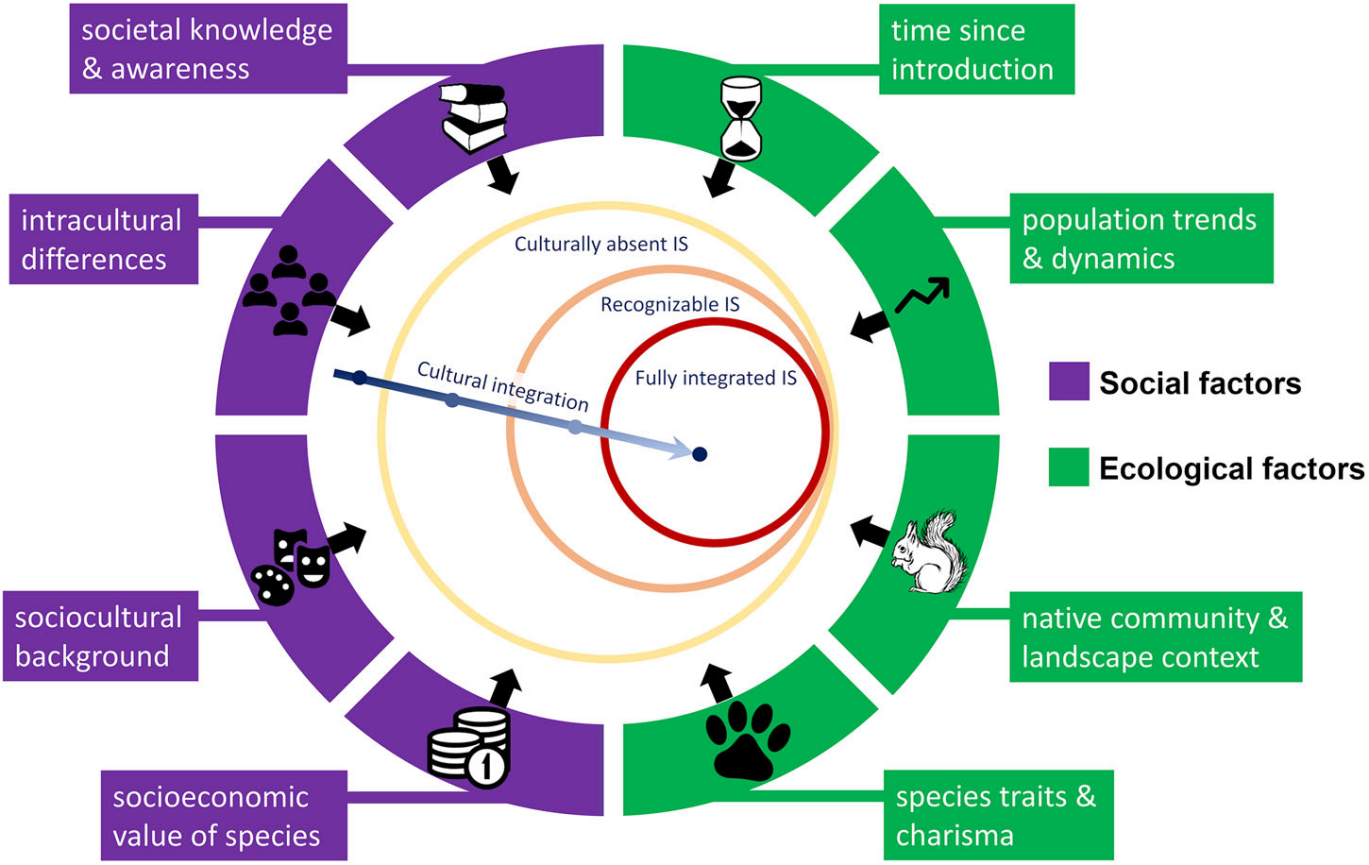
The process and key factors affecting cultural integration of invasive non-native species (IS). Following its introduction, an invasive non-native species can either remain culturally absent or enter the cultural sphere, where it can over time become fully integrated and perceived as being native, and an integral or even essential part of culture. Fields surrounding the central diagram present the key factors affecting the cultural integration process.

Various factors influence the process of cultural integration of invasive species, including social factors (societal knowledge and awareness, intracultural differences, sociocultural background, and instrumental, intrinsic and relational values ascribed to the species)^47^ and ecological factors (time since introduction, population size and dynamics, native community and landscape context, and species traits; Fig. 3)^7,37,41,46,48^. Processes and forms of cultural integration and the pace at which they unfold vary spatially and temporally, across and within societies^7,41,48^. The main factors highlighted in the literature are presented below.

### Time since introduction

There is likely a strong relationship between the time since the invasive species’ introduction to a new region and its cultural integration. People’s ability to identify species as non-native or invasive changes with time. A species introduced in the distant past has had more time to become embedded in the local culture and memory^7,30,41^. For example, bluegrass (*Poa pratensis*) and tumbleweed (*Kali tragus*), introduced to the USA from Eurasia, have come to be seen as natural and iconic landscape elements in the “Bluegrass region” and the American West, respectively, with very few people being aware of their non-native origin^24,49^. The process is often associated with changes in the names of introduced species. Over time, introduced species can experience further evolutionary changes, driven by the selective pressure in their novel range^50^. Ultimately, they can undergo speciation, which can further affect their taxonomic, cultural, or non-native status^51^.

### Population status, abundance, dynamics and spread

Population status, abundance, and spread of invasive species can strongly affect the cultural integration process. Overall, cultural salience tends to be higher for more abundant species with wider geographic ranges that have a higher overlap with human populations^34,52^. This leads to greater visibility of such species and provides greater opportunity for interactions with humans and the formation of affective attachments^18,53^ (Boxes 1 and 2). The process of integration can also be affected by changes in the invasive species ecology, population structure, or dynamics that may affect its salience, appeal, or perceived value. For example, large-scale expansions and associated impacts can over time shift positive perceptions to negative ones^46^.

### Species traits, life history, and charisma

Certain invasive species traits can facilitate cultural integration, namely those that drive their perceived appeal or charisma^37^ or benefits/usefulness^54^ (see below). Charismatic invasive species often become culturally significant or iconic even if their non-native and invasive status is still widely recognized, such as feral hippopotamuses (*Hippopotamus amphibius*) in Colombia, Chinese windmill palm (*Trachycarpus fortunei*) in Switzerland, and Jacaranda trees (*Jacaranda mimosifolia*) in South Africa, which became a symbol of the city of Pretoria^37,41,55^. The ruddy duck (*Oxyura jamaicensis*), introduced in UK, was also adopted as the emblem of the birdwatchers’ club in the area of its first introduction^37^. Invasive species that exhibit unique features, such as trees of large stature or conspicuous flowers, tend to be highly valued by people, irrespective of their origin^16,19,56^. Overall, species that can build or strongly affect their invaded ecosystems, such as foundation species and ecosystem engineers, may become more quickly associated with the new environment and landscapes. For example, the groves formed by *Eucalyptus* species introduced to California in the 19th century have come to be appreciated as a characteristic feature of the state’s landscapes^6^. Similarly, after invading large arid areas in Spain, agave (*Agave spp*.) and prickly pear cacti (*Opuntia spp*.) have become iconic symbols of the landscape, while negatively impacting communities of native species^37^.

### Native community and the landscape context

Morphological similarity between invasive and native species can facilitate their cultural integration. At the same time, similarity among species may lead to a stronger overlap of their respective cultural niches and to stronger cultural competition and cultural replacement of native species (Fig. 2). Such processes can be strengthened by their biological interactions, for example, if invasive species also drive population or range reduction of native species, replacing them both biologically and culturally^24^. For instance, the replacement of native turtle species in Japan by the invasive red-eared slider (*Trachemys scripta* subsp. *elegans*) may have contributed to shifting baselines in people’s turtle awareness and knowledge manifested in cultural products such as toys^24^ (Fig. 1B). On the other hand, invasive species without an analogous, morphologically similar native species, can also become integrated owing to their novelty and perceived uniqueness or charisma^37^. Invasive species can fill an empty ecological niche in an ecosystem, or present novel uses to people, which makes them more readily accepted and valued by local communities. For example, Australian *Acacia* tree species (wattles) have become culturally important for poor rural communities living in grassland areas in South Africa with limited access to electricity, as these trees provide a scarce resource (fuelwood) in this tree-limited and structurally underdeveloped landscape context^46^.

The cultural identity of invasive species can be transferred from extant or extirpated native species. Such a process occurs unconsciously and often through taxonomic misidentification, for example, if the two species are not easily distinguishable morphologically, as is the case for native and invasive species of tilapia in the Sea of Galilee, represented by a complex mixture of their populations, as well as their hybrids^57^.

### Societal knowledge and awareness

Lack of local knowledge about invasive species, native biodiversity, the invasion phenomenon in general, and its impacts, can facilitate cultural integration^43,58,59^. For example, a survey in the sub-Antarctic Magellanic ecoregion of South America showed that the awareness of local communities regarding the surrounding flora was not dominated by native plants but by non-native, cosmopolitan ornamental species, likely driven by media and their everyday encounters with these species in urban areas^60^. High awareness of invasive species’ negative impacts such as threats to the economy or human health often hinder their integration. Nevertheless, even negatively perceived invasive species do at times become culturally integrated, such as the invasive cane toad (*Rhinella marina*) in Australia, which has become for some a symbol of resilience, adaptability, and transformation^61^.

### Species cultural uses, benefits, and values

Cultural integration is more likely if invasive species are considered beneficial to local livelihoods or the economy^37,62,63^. Such benefits can include regulating services (e.g., pollination), material/provision services (e.g., medicine or raw material), and non-material/cultural services (e.g., recreation or learning)^8,37,43,48^. The use of invasive species as a food source is a powerful driver of cultural integration^4^. For example, following their introduction worldwide for food and hunting, feral pigs (*Sus scrofa*) have become strongly associated with local cultures, including traditional, subsistence, and recreational hunting, as well as traditional cuisine^31,64^ (Fig. 1C). Economic value represents one of the main drivers of intentional introductions^6^. Furthermore, management programs based on creating a market for invasive species use can contribute to their cultural integration, by enhancing their economic value through marketing efforts^4^.

### Sociocultural background

Several sociocultural factors affect cultural integration, including people’s relationship with and access to nature, existing value systems, general perceptions of biodiversity and the environment, environmental governance contexts and the level of cultural insularity, globalization, and urbanization^7,16,41,65^. Cultural integration is further influenced by sociocultural changes in society, including people’s growing disconnection from nature^40^, the ongoing erosion of Indigenous and local knowledge systems^66^, as well as the rise of biophobia (fear of nature)^67^.

Cultural integration can also be affected by people’s origins or their movement^41^. For example, immigrants from Europe and Asia settling in the Americas and the Pacific deliberately introduced non-native species that were considered culturally and socioeconomically relevant at their place of origin^6,19,68,69^. This was organized through so-called acclimatization societies. It was mainly done for esthetic or economic reasons, as well as for psychological support, by attempting to recreate a familiar environment and regain a sense of place and continuity for colonial or immigrant communities^6,7,19,31,70^.

### Intercultural and intracultural differences

Along with the broader cultural and societal patterns described above, finer-grained perceptions and attitudes towards invasive species vary across specific groups, social sectors, and stakeholders^7,30,31^. For example, *Echium plantagineum* is called “salvation Jane” for its value to beekeepers and dryland graziers in Australia and “Patterson’s curse” by those producing crops, reflecting different perceptions of this invasive plant species from Europe^16^. Similarly, invasive fish species can be simultaneously perceived as a promising opportunity by some recreational fishers but negatively by others^71,72^. Perceptions are also affected by differing religious norms and ethical value systems within different cultural groups^7^. Such differences within a society can make the speed and outcome of the cultural integration process highly complex and partly unpredictable^8,41^. In these instances, relational frameworks that unpack values can lead to a better understanding of potential social and ecological futures that are possible, desirable, and just, for ecosystems that include invasive species^13,72^.

## Practical implications and impacts of cultural integration

### Impacts on management

Human relationships with invasive species are complex and multi-faceted, and challenge the binary concepts of invasive species as being either culturally impoverishing (‘bad’) or enriching (‘good’)^8^. Non-native species can provide benefits to many people when they are fully incorporated into cultures and livelihoods; however, at the same time, they can cause large-scale negative social-ecological impacts and challenges^48,73^. The process of integration can lead to public opposition to management and generate unanticipated social conflicts (where some actors may benefit, while others are harmed by the species)^31,73^. For example, the public is typically less supportive of harsher management actions, such as culling, for species perceived as native or desirable, especially if they are considered charismatic or iconic, or have use values^37,74,75^.

Invasive species that have acquired sociocultural or economic value that exceeds their perceived negative impacts might paradoxically, from the perspective of conservation specialists, be subjected to protection or restoration measures^4,28,37,49^. Moreover, in cases where invasive species become more valued than native species, such measures may run in parallel with the control of native species to mitigate their competition with the invasive species and promote the invasion process. Examples of such paradoxical scenarios include the poisoning of native guanacos (*Lama guanicoe*) in Patagonia to reduce competition with invasive red deer (*Cervus elaphus*) and livestock^9^, promotion of the invasion of the cattail (*Typha domingensis*) by local communities in Mexico at the expense of the native California bulrush (*Schoenoplectus californicus*; Fig. 1D)^76^, and the active spread of the culturally valuable but highly invasive Nypa tree (*Nypa fruticans*) in Nigeria^77^. Cultural integration can also stimulate intentional invasive species introductions and thus contribute to secondary introductions and further spread.

### Effects on human culture

Cultural integration of invasive species can affect people’s perceptions of their environment, their values, traditions, and customs, modify collective memory, and even alter historical knowledge and understanding^6,16,43^. Consequently, cultural integration can lead to fundamental societal changes. For example, the integration of invasive prickly pear cactus species (*Opuntia* spp.) in Madagascar contributed to a shift within local communities from mobile pastoralism to settled agricultural practices^54^.

Just as biological invasions lead globally to the homogenization of biological diversity, they can have the same effect on cultural diversity (but see discussion below about potential positive effects). Through this process, also termed “biocultural homogenization”^60^, the cultural presence of invasive species suppresses the cultural presence and identities of native species and their associated cultural services, and ultimately leads to their societal extinction^8,34,78,79^.

When the process of integration affects culturally important native species that play key roles in supporting cultural identity and social cohesion^80^, it leads to the restructuring of sociocultural systems or the establishment of distinct, novel social-ecological systems^8,79,81^. Such changes can be gradual, but they may ultimately lead to social-ecological tipping points and associated social-ecological regime shifts^82,83^ with irreversible changes to local social systems, which increase societal vulnerability and reduce peoples’ resilience (e.g., health, job security, poverty, income). This, for example, happened with biological invasions in Lake Victoria, where impacts of invasive species such as water hyacinth (*Eichhornia crassipes*) and Nile perch (*Lates niloticus*) led to massive ecosystem transformations and strong shifts in social-ecological systems, with complex effects on job opportunities, industry, infrastructure, and land uses in the wider watershed^81,83^.

### Specific impacts for Indigenous Peoples and local communities

Indigenous Peoples, small-holders and traditional knowledge holders are disproportionately affected by social and environmental changes and globalization^66,84^. This can make them particularly vulnerable to the impacts of biological invasions, as the process of cultural integration of invasive species may additionally impact culturally important species and consequently negatively affect societies and knowledge systems that depend on them. Many Indigenous Peoples have already experienced such changes, with widespread shifts from using native species to invasive species in their livelihoods and traditions^7,25,27^. Over time, this can lead to potentially irreversible negative changes and exacerbate existing pressures on traditional ecological knowledge and biocultural heritage associated with native species and communities^14,66^.

On the other hand, there is often a high level of awareness among Indigenous Peoples and other traditional communities of invasive species and their potential social-ecological impacts^25,27,85,86^. Several scholars argue that invasive species management should apply a biocultural lens to align more closely with Indigenous land-based stewardship^27,87,88^. Indeed, biocultural frameworks that acknowledge the inextricable inter-connections between biodiversity, language, and culture, see humans as part of nature, and focus on relationships between humans and other species. Such frameworks are becoming a major area of scholarship within applied ecology, ethnobiology, and related disciplines, and gain traction as an effective, just, and culturally-appropriate model for invasive species management^28,89^.

### Positive societal effects of cultural integration

Cultural integration can also lead to a wide range of positive effects for people, for example, by strengthening attachments to nature, strengthening or developing new human–nature interactions, providing important resources, promoting food security, and enriching local cultures. Invasive species enrich cultures through the positive role they can play in livelihoods, traditions, spirituality, inspiration, and local cultural identities^8,43,49,78^. For example, some local communities in Australia have established spiritual associations with invasive species, such as dromedary camels (*Camelus dromedarius*)^31^. Invasive species are more likely to become “culturally enriching”^8^ when they occupy a cultural space that was previously vacant, also known as a substitute (Fig. 2).

### Broad sustainability implications

Cultural integration of invasive species brings forth both challenges and opportunities to achieve sustainability goals. It can act as a barrier to sustainability transitions, for example, by negatively affecting social justice and intergenerational equity through impoverishment and homogenization of cultural and biological diversity, causing conflicts and impeding control actions. It can obstruct the long-term stability of social-ecological systems by influencing and modifying place-based human–nature relationships that have supported them^80^. Such impacts might particularly threaten Indigenous Peoples by impairing their knowledge systems and livelihoods, which represent the backbone of their identity and survival^90^. On the other hand, cultural integration can help promote the resilience of social-ecological systems by, for example, improving food sovereignty and security.

Acknowledging the cultural integration of invasive species can improve our understanding of the societal aspects of biological invasions, as well as the social and cultural dimensions of sustainability transitions, which can help guide effective and equitable governance and management implementation. However, fully addressing the different aspects of cultural integration of invasive species within sustainability science and practice will require full recognition of the complexity of this process through adequate changes in policy and practice. For example, social systems and cultures tend to adapt to biological invasions at different rates than ecosystems^42^. Consequently, management plans need to be designed to work across a wide range of temporal and spatial scales^43^. Furthermore, the diversity of perceptions within society can only be adequately incorporated in sustainability management through participatory governance.

## Mitigation and adaptation strategies

The consequences of the cultural integration of invasive species need more attention in research, education, decision-making, policy, and management, with adequate involvement of all sectors of society and relevant stakeholders, especially when they are in part or entirely harmful to biological and cultural diversity. Invasive species management and decision-making need to be science-based, socially inclusive, and participatory to the largest extent possible^44^, with open and fair involvement to ensure diverse perspectives and strengthen trust in and societal support for the process^31,54,91^. Potential conflicts can be mitigated by involving stakeholders and rights holders early in the management phase, gathering their first-hand knowledge and perspectives, and seeking solutions based on consensus among environmental, social, and economic priorities^32,73,76,92^.

Invasion science, management, and policy recently started to shift from a dominantly biological focus to a transdisciplinary perspective^93^. Such a shift will benefit from expertise and insights of a wide range of disciplines and stakeholders to capture the complexity of sociocultural processes associated with biological invasions^7,8,31^. Yet, implementing a truly holistic perspective of social-ecological systems will require closer involvement of social sciences and humanities^43,94^, as well as perspectives outside of academia^95^. The cases reviewed in this article exemplify many of the different ways in which biocultural relations shape people’s understandings of their roles within and responsibilities towards their environment, including invasive species^88^. Biocultural approaches should therefore be recognized as an essential prism to look at the interwoven relationships between people and invasive species from culturally grounded perspectives^28,89^. Such an approach can recognize, at times, a mismatch between the cultural acceptance of the non-native species by various place-based communities and its ecological-scientific perception in the eyes of scientists and nature conservationists.

Culturally integrated invasive species that have negative impacts can be managed by changing cultural relationships, for example, through education and awareness raising. Such efforts can be directed to improve awareness and literacy regarding invasive species^17^, including knowledge and recognition of the invasion process and impacts^6,41^. Management should simultaneously improve knowledge of native species potentially affected— their threat status, and ecological and sociocultural importance. Such a process can further strengthen the “sense of place” and human–nature interactions, and stimulate interest in biodiversity and sustainability^17^.

However, obstructing or reversing the process of cultural integration is probably not always appropriate or advisable. In some specific situations, the removal of invasive species can lead to considerable and unforeseen negative biological effects^96,97^, while the disruption of their sociocultural embeddedness can lead to a wide range of impacts on culture, livelihoods, and the economy^54,64^. Biological invasions have played an important role throughout history in shaping and enriching human culture^31,43^. Past reference conditions are not always viable or even desirable restoration goals in systems that have undergone dramatic environmental, social, and cultural changes. Both environmental and social justice need to be strongly considered where cultural integration has occurred^98^. For example, management to remove culturally integrated invasive species may disproportionately affect Indigenous Peoples and local communities who rely on them and have incorporated them into cultural practice. This relates to the multiple effects of colonization on Indigenous lands and people, including, but not limited to, their removal from lands and deportation to unfamiliar locations. A collaborative and equitable decision-making process with these communities is therefore essential to successfully remove invasive species without perpetuating feelings of injustice^98^. Any potential action to hinder or reverse the process of cultural integration should thus carefully evaluate biological, socioeconomic, and cultural costs and benefits through a participatory, power-sharing approach.

The cultural integration of invasive species may be not only desirable but even necessary for some management issues, such as managed relocation efforts^99^, for example, as a climate-change mitigation measure^100^, or in the case of ongoing, unmanaged distributional range shifts due to climate change (neonatives or “species on the move”)^101,102^. Cultural integration is also important in the case of reintroductions or rewilding with substitute species^103^, particularly for those species that were never culturally present or have been lost from collective memory^34^. For example, the reintroduction of the Eurasian beaver (*Castor fiber*) in Central Europe has led to conflicts with local communities, mainly because many such communities often perceive it as a pest rather than a lost-and-reintroduced natural element of the environment^104^.

The process of cultural integration of invasive species and its effects, both positive and negative, should be recognized and incorporated into existing frameworks, such as the values-based decision framework^32^, the mitigation hierarchy^105^, and other promising concepts such as invasion syndromes^106^. However, a major challenge will be to find a way to assess the complexity of societal values related to the process of cultural integration of invasive species. One potential approach in this respect could be Turner’s multidimensional index of the cultural significance of a species, based on estimates of the quality, intensity, and exclusivity of species use^107^ and ensuring equitable decision-making on the future of invasive species with communities affected. Another promising approach could be the biocultural indicator for sustainability by Sterling et al.^108^, which can capture both ecological and social-cultural factors across local and global initiatives to increase resilience of both humans and ecosystems, as well as account for their linkages and feedback. Furthermore, frameworks such as EICAT, EICAT+ and SEICAT can provide information on the magnitude of both positive and negative impacts, including social aspects^109–111^; these frameworks can be complemented by approaches more strongly embedded in social sciences, or by exploring new cultural values arising from cultural integration.

To ensure the long-term sustainability of social-ecological systems, ongoing biocultural changes will need to be addressed through transdisciplinary research and participatory governance and management, based on inclusion, equity, justice, and open, responsive communication^73,112^. Ultimately, future research and management efforts that focus on the cultural integration of invasive species will have to fully recognize that biological invasions are as much a sociocultural phenomenon as they are a biological one.

## Supplementary information

Supplementary Information

## Data Availability

No datasets were generated or analysed during the current study.
