# Supplementary material for: Cultural integration of invasive species
Source: NPJ Biodivers. 2025 Jun 26;4:25. doi: 10.1038/s44185-025-00097-3 (PMC12202807; doi:10.1038/s44185-025-00097-3)
Supplement: Supplementary file 1 — Supplementary Information [file 44185_2025_97_MOESM1_ESM.pdf]

I. Jarić *et al*. – Supplementary Table 1. Glossary.

Term	Definition
Behavioural interventions	Measures aimed at encouraging societally valued behaviour change[1].
Biophobia	Fear of nature, negative feelings or responses to certain natural stimuli[2].
Cultural identity	Subjective identification with a particular cultural group[3].
Cultural integration	A process whereby non-native species gradually become embedded within the local culture, becoming perceived by the public as familiar, native elements of the environment, and/or as an integral part of local culture.
Cultural keystone species	Culturally salient species that strongly shape the cultural identity of people, as reflected in the fundamental roles these species have in diet, materials, medicine, and/or spiritual practices[4].
Cultural niche	Those parts of the human cultural environment that a species occupies[5].
Cultural product	Tangible and intangible creations of a particular culture.
Extant species	A species that has survived to the present day.
Extirpated species	A locally extinct species, which has disappeared from a certain geographical delimitation.
Invasive non-native species	Organisms known to have established and spread with negative impacts on biodiversity, local ecosystems and species. Many invasive species also affect nature's contributions to people (embodying different concepts such as ecosystem goods and services, and nature's gifts) and good quality of life[6].

Nature	All living organisms and ecosystems, excluding those that are not self-sustained[7].
Non-native taxon	A species, subspecies, or lower taxon occurring outside of its natural range (past or present) and dispersal potential (i.e. outside the range it occupies naturally or could not occupy without direct or indirect introduction or care by humans), including any part, gametes or propagule of such species that might survive and subsequently reproduce. Also known as non-native, non-indigenous, foreign, or exotic species[6].
Ruderal species	Species that are first to colonize disturbed lands, i.e. lands laid bare by natural events, such as wildfires, or human action, such as construction or agriculture.
Shifting baseline syndrome	A gradual change in the accepted norms for the condition of the natural environment due to lack of past information or lack of experience of past conditions[8].
Societal extinction of species	Loss of societal attention and collective memory of a species[9].
Societal salience of a species	Cultural profile and visibility, or public popularity of a species.
Species on the move	Species whose distributions are shifting in response to climate change[10].
Vicarious experiences	Indirect, disembodied experiences, based on virtual exposure to species, through various physical or digital records from the literature, arts, oral traditions, or media[11].

References

1. Balmford, A. et al. Making more effective use of human behavioural science in conservation interventions. *Biol. Conserv.* **261**, 109256 (2021).

2. Soga, M., Gaston, K. J., Fukano, Y. & Evans, M. J. The vicious cycle of biophobia. *Trends Ecol. Evol.* **38**, 512-520 (2023).

3. Usborne, E. & De La Sablonnière, R. Understanding my culture means understanding myself: The function of cultural identity clarity for personal identity clarity and personal psychological well-being. *J. Theory Soc. Behav.* **44**, 436-458 (2014).

4. Garibaldi, A. & Turner, N. Cultural keystone species: implications for ecological conservation and restoration. *Ecol. Soc.* **9**, 1 (2004).

5. Schuetz, J. G. & Johnston, A. Tracking the cultural niches of North American birds through time. *People Nat.* **3**, 251-2 (2021).

6. IPBES. Summary for policymakers of the thematic assessment report on invasive alien species and their control of the Intergovernmental Science-Policy Platform on Biodiversity and Ecosystem Services (eds. Roy, H. E. et al.). IPBES secretariat, Bonn, Germany (2023).

7. Soga, M. & Gaston, K. J. The dark side of nature experience: Typology, dynamics and implications of negative sensory interactions with nature. *People Nat.* **4**, 1126-1140 (2022).

8. Soga, M. & Gaston, K. J. Shifting baseline syndrome: causes, consequences, and implications. *Front. Ecol. Environ.* **16**, 222-230 (2018).

9. Jarić, I. et al. Societal extinction of species. *Trends Ecol. Evol.* **37**, 411-419 (2022).

10. Pecl, G. T. et al. Climate-driven ‘species-on-the-move’ provide tangible anchors to engage the public on climate change. *People Nat.* **5**, 1384-1402 (2023).

11. Gaston, K. J. & Soga, M. Extinction of experience: The need to be more specific. *People Nat.* **2**, 575-581 (2020).
